# Data on the coefficient of friction and its prediction by a machine learning model as a function of time for open-cell AlSi10Mg-Al_2_O_3_ composites with different porosity tested by pin-on-disk method

**DOI:** 10.1016/j.dib.2023.109489

**Published:** 2023-08-10

**Authors:** Mihail Kolev, Ludmil Drenchev

**Affiliations:** Institute of Metal Science, Equipment and Technologies with Center for Hydro- and Aerodynamics “Acad. A. Balevski”, Bulgarian Academy of Sciences, 1574 Sofia, Bulgaria

**Keywords:** Al-based metal matrix composites, Coefficient of friction, Extreme gradient boosting model, Machine learning

## Abstract

This data article presents the experimental data of the wear behavior of four types of open-cell AlSi10Mg materials and open-cell AlSi10Mg-Al_2_O_3_ composites with different pore sizes under dry sliding conditions tested by pin-on-disk method. The data include the coefficient of friction (COF) as a function of time for each material, as well as the predictions of COF using a machine learning model - Extreme Gradient Boosting. The data were generated to investigate the effect of pore size and reinforcement on the friction and wear properties of open-cell AlSi10Mg-Al_2_O_3_ composites, which are promising materials for lightweight and wear-resistant applications. The data can also be used to validate theoretical models or numerical simulations of wear mechanisms in porous materials, as well as to optimize the material design and processing parameters to enhance the wear resistance of open-cell AlSi10Mg materials. The data are available in DWF and XLSX format and can be opened by any text editor or spreadsheet software. The data article is related to an original research article entitled “Production and Tribological Characterization of Advanced Open-Cell AlSi10Mg-Al_2_O_3_ Composites”, where the details of the experimental methods, the microstructural characterization, and the analysis of the wear mechanisms are provided [1].

Specifications Table

**Table:** 

Subject	Materials Science
Specific subject area	Surface Science and Engineering Tribology
Type of data	XLSX files DWF files TXT files PNG files Python programming code (.py) Table Figure
How the data were acquired	Dry wear tests performed on four types of open-cell AlSi10Mg materials and open-cell AlSi10Mg-Al_2_O_3_ composites with different pore sizes and reinforcements using Ducom Rotary (Pin/Ball-on-Disk) tribometer, model TR-20 Ducom at room temperature with linear velocity 0.5 m·s^−1^, load 50 N and sliding time of 420 s.
Data format	Raw Processed
Description of data collection	The data were collected from dry wear tests performed on four types of open-cell AlSi10Mg materials with different pore sizes and reinforcements. The materials were fabricated by liquid-state processing route by the replication method. The wear tests were conducted using a pin-on-disk tribometer at room temperature with linear velocity 0.5 m.s^−1^, load 50 N and time of 420 s. The specimens were cylindrical pins with diameter 10 mm and length 20 mm. The counterface was a hardened steel disk with hardness 62 HRC and roughness Ra 1.4 µm. The COF was determined as the ratio of the friction force to the applied load. The COF was recorded as a function of time for each specimen during the tests. The predictions of COF were made using an Extreme Gradient Boosting model.
Data source location	Institution: Institute of Metal Science, Equipment and Technologies with Center for Hydro- and Aerodynamics “Acad. A. Balevski” at Bulgarian Academy of Sciences. City/Town/Region: Sofia 1574, 67 “Shipchenski prohod” str. Country: Bulgaria. Latitude and longitude: 42.67836627128253, 23.368534487637326
Data accessibility	Repository name: data.mendeley.com Data identification number/DOI: 10.17632/2356m76ktj.1 Direct URL to data: https://data.mendeley.com/datasets/2356m76ktj/1 Instructions for accessing the data: Anyone can access the data from the direct URL of the repository, the data do not have any special restrictions or access controls.
Related research article	Kolev, M.; Drenchev, L.; Petkov, V.; Dimitrova, R. Production and Tribological Characterization of Advanced Open-Cell AlSi10Mg-Al_2_O_3_ Composites. Metals 2023, 13, 131. https://doi.org/10.3390/met13010131.

## 1 Value of the Data

• These data are useful for understanding the wear behavior of open-cell AlSi10Mg materials and open-cell AlSi10Mg-Al_2_O_3_ composites with different pore sizes under dry sliding conditions.
• Researchers and engineers who are interested in developing lightweight and wear-resistant materials for various applications such as sliding contact bearings, where low friction and high load-bearing capacity are required, can benefit from these data.
• The experimental results can be compared with theoretical models or numerical simulations of wear mechanisms in porous materials.
• The effect of pore size and reinforcement on the friction and wear properties of open-cell AlSi10Mg- Al_2_O_3_ composites can be analyzed using these data.
• The performance of the machine learning model for predicting the coefficient of friction as a function of sliding time for different materials can be evaluated using these data.
• The possibility of optimizing the material design and processing parameters to enhance the wear resistance of open-cell AlSi10Mg materials can be explored using these data.

## 2 Objective

The objective of this data article is to present the experimental data of the wear behavior of four types of open-cell AlSi10Mg materials with different pore sizes and reinforcements under dry sliding conditions. The data include the coefficient of friction (COF) as a function of time for each material, as well as the predictions of COF using the machine learning model - Extreme Gradient Boosting (XGBoost). The data were generated to investigate the effect of pore size and reinforcement on the friction and wear properties of open-cell AlSi10Mg materials, which are promising materials for lightweight and wear-resistant applications. The data can also be used to validate theoretical models or numerical simulations of wear mechanisms in porous materials, as well as to optimize the material design and processing parameters to enhance the wear resistance of open-cell AlSi10Mg foams. This data article is related to an original research article [1], where the details of the experimental methods, the microstructural characterization, and the analysis of the wear mechanisms are provided. The data article adds value to the published article by making the raw and processed data publicly available and reusable for further research and development.

## 3 Data Description

The data presented in this article are related to the wear behavior of four types of open-cell AlSi10Mg materials with different pore sizes. The materials are:

• AlSi10Mg with Al_2_O_3_ as reinforcement and pore size of 800–1000 µm;
• AlSi10Mg without reinforcement and pore size of 800–1000 µm;
• AlSi10Mg with Al_2_O_3_ as reinforcement and pore size of 1000–1200 µm;
• AlSi10Mg without reinforcement and pore size of 1000–1200 µm.

The data were collected by performing dry wear tests at room temperature with linear velocity 0.5 m·s^−1^, load 50 N and sliding time of 420 s. Each label of the specimen corresponds to three datasets from pin-on-disk tests with open-cell AlSi10Mg-Al_2_O_3_ composite (AC; AE) and open-cell AlSi10Mg material (C; E) with different pore sizes. The labels and their corresponding pore sizes are:

• AC_3_2, AC_3_3, AC_3_4 (pore size between 800 and 1000 µm);
• C_5_1, C_5_2, C_5_3 (pore size between 800 and 1000 µm);
• AE_3_2, AE_4_1, AE_6_6 (pore size between 1000 and 1200 µm);
• E_3_1, E_6, E_6_3 (pore size between 1000 and 1200 µm).

The data folders and files are stored organized in the in Mendeley Data repository. The repository consists of two main folders: “Pin-on-disk_data” and “Prediction”. The “Pin-on-disk_data” folder has three subfolders that store the raw, processed, and average data of the COF as a function of time for each of the four tested materials, as well as the plots and the python script for the COF calculation. The “Prediction” folder has three subfolders that store the input data, the output data, and the python script for the predictions of COF vs sliding time using a XGBoost model. The output data includes the actual and predicted values of COF for two sets (test and validation) of each material, the performance metrics of the predictions, and the plots of the actual vs predicted COF as a function of time for each material.

1) Pin-on-disk_data folder contains three subfolders (Raw_files; Processed_files; COF_calculation). The files stored in the Raw_files subfolder are:
  • “AC_3_2.dwf”: Data for AC_3_2 specimen;
  • “AC_3_3.dwf”: Data for AC_3_3 specimen;
  • “AC_3_4.dwf”: Data for AC_3_4 specimen;
  • “C_5_1.dwf”: Data for C_5_1 specimen;
  • “C_5_2.dwf”: Data for C_5_2 specimen;
  • “C_7_1.dwf”: Data for C_7_1 specimen;
  • “AE _3 _2.dwf”: Data for AE _3 _2 specimen;
  • “AE _4 _1.dwf”: Data for AE _4 _1 specimen;
  • “AE _6 _6.dwf”: Data for AE _6 _6 specimen;
  • “E _3 _1.dwf”: Data for E _3 _1 specimen;
  • “E _6.dwf”: Data for E _6 specimen;
  • “E _6 _3.dwf”: Data for E _6 _3 specimen;

The files stored in the Processed_files subfolder are:

• “AC_3_2.xlsx”: Data for AC_3_2 specimen;
• “AC_3_3.xlsx”: Data for AC_3_3 specimen;
• “AC_3_4.xlsx”: Data for AC_3_4 specimen;
• “C_5_1.xlsx”: Data for C_5_1 specimen;
• “C_5_2.xlsx”: Data for C_5_2 specimen;
• “C_7_1.xlsx”: Data for C_7_1 specimen;
• “AE_3_2.xlsx”: Data for AE _3 _2 specimen;
• “AE _4 _1.xlsx”: Data for AE _4 _1 specimen;
• “AE _6 _6.xlsx”: Data for AE _6 _6 specimen;
• “E _3 _1.xlsx”: Data for E _3 _1 specimen;
• “E _6.xlsx”: Data for E _6 specimen;
• “E _6 _3.xlsx”: Data for E _6 _3 specimen;

The files stored in the COF_calculation subfolder are:

• “Average Coefficient Of Friction For Material AC.xlsx” and “Average Time For Material AC.xlcx”: Output from a python script average data COF and time data of all tested specimens (AC _3 _2; AC _3 _3; AC _3 _4).
• “Average Coefficient Of Friction For Material C.xlsx” and “Average Time For Material C.xlcx”: Output from a python script average data COF and time data of all tested specimens (C _5 _1; C _5 _2; C _7 _1).
• “Average Coefficient Of Friction For Material AE.xlsx” and “Average Time For Material AE.xlcx”: Output from a python script average data COF and time data of all tested specimens (AE _3 _2; AE _4 _1; AE _6 _6).
• “Average Coefficient Of Friction For Material E.xlsx” and “Average Time For Material E.xlcx”: Output from a python script average data COF and time data of all tested specimens (E _3 _1; E _6; E _6 _3).
• “figure9.png” and “figure10.png”: Python script using the matplotlib library for visualization of the average COF vs time for materials (“figure9.png” - AC and C) and (“figure10.png” - AE and E).
• “calculate-wear-Al-Al2O3.py”: Python script for visualization of the average COF vs time, storing the two output plots in PNG and storing the files in XLSX of the average COF and average time for all materials, based on the input of the obtained from the wear tests datasets of friction force and time.

Prediction folder contains three subfolders (Input_files; Output_data; Python_COF_prediction). The files stored in the Input_files subfolder are:

• “pred_COF_AC.xlsx”, “pred_COF_C.xlsx”, “pred_COF_AE.xlsx”, and “pred_COF_E.xlsx”: Used as input files for the python script to load the data from each of the files and make the predictions.

The files stored in the Output_data subfolder are:

• “test_val_data_C.xlsx”, “test_val_data_AC.xlsx”, “test_val_data_E.xlsx”, “test_val_data_AE.xlsx”: stored data of the actual and predicted values of the COF for two different sets (test and validation) of the four materials.
• “AC_performance_metrics.txt”, “C_performance_metrics.txt”, “AE_performance_metrics.txt”, “E_performance_metrics.txt”: Calculated and stored in four files for the four materials the performance metrics of the predicted COF.
• “pred_COF_AC.png”, “pred_COF_C.png”, “pred_COF_AE.png”, and “pred_COF_E.png”: Stored plot as a PNG of the average actual vs predicted COF as a function of time.

The file stored in the Python_COF_prediction subfolder is:

• “XGB-COF.py”: Python script used for visualizing and storing a PNG file with the average actual COF vs the predicted as a function of time, calculating and storing in a TXT file the performance metrics of the predicted COF, and storing in a XLSX file the data used for the visualization of the average actual COF.

**Fig. 1:**
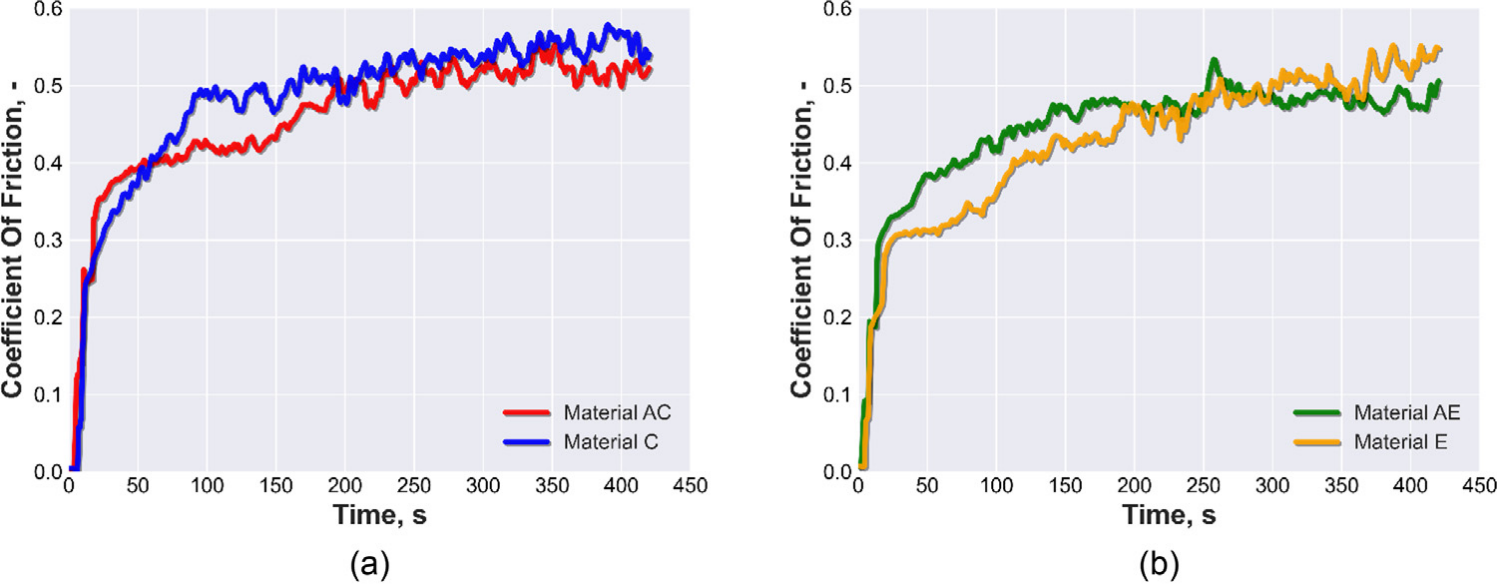
Average COF vs time obtained by pin-on-disk tests with linear speed 0.5 s^−1^, load of 50 N and sliding time of 420 s: (a) of AC and C; (b) of AE and E.

**Fig. 2:**
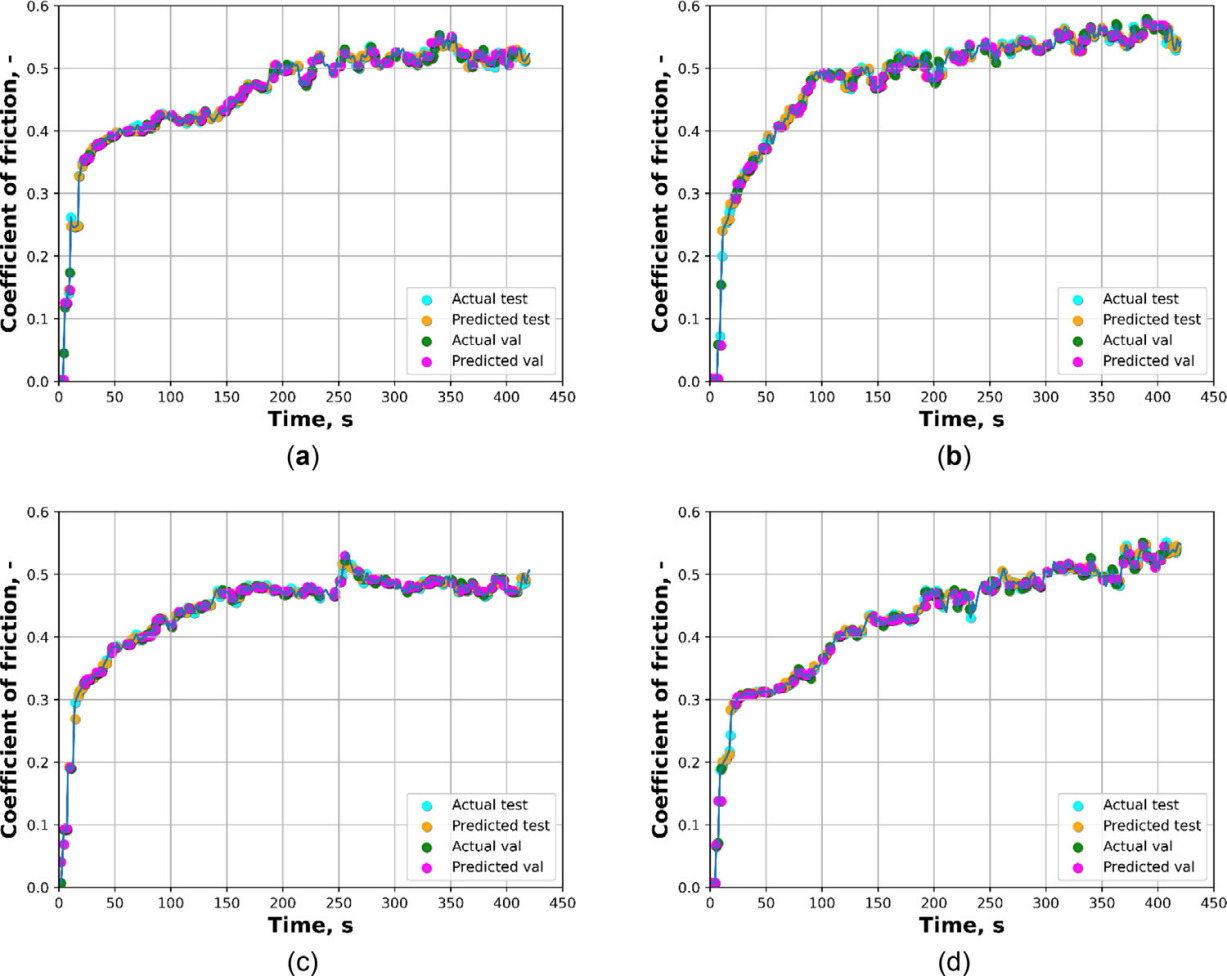
Average actual vs predicted coefficient of friction as a function of sliding time for the following materials: (a) AC; (b) C; (a) AE; (b) E.

Fig. 1a presents the average COF vs time for materials AC and C with pore size 800–1000 µm. Fig. 1b shows the average COF vs time AE and E with pore size 1000–1200 µm. Both figures in Fig 1 were created by a python script included in the repository “calculate-wear-Al-Al2O3.py”. Fig. 2 shows four plots of the average actual vs predicted COF as a function of time for the materials AC, C, AE, and E. All figures presented in Fig 2 were created by a python script included in the repository “XGB-COF.py”. The XGBoost model calculated the performance metrics of the test-set and validation-set for the four materials, which include mean squared error (MSE), root mean squared error (RMSE), coefficient of determination (R2), and mean absolute error (MAE), as shown in Table 1. Table 2 shows the descriptive statistics of the average COF for all materials, including the mean, median, standard deviation, minimum and maximum values.

**Table 1:** Test-set and validation-set performance metrics.

Material	Set	R2	MAE	MSE	RMSE
AC	Test	0.9971	0.0037	0.0000	0.0048
AC	Validation	0.9934	0.0050	0.0001	0.0082
C	Test	0.9944	0.0052	0.0001	0.0078
C	Validation	0.9875	0.0068	0.0002	0.0137
AE	Test	0.9936	0.0040	0.0000	0.0063
AE	Validation	0.9965	0.0035	0.0000	0.0056
E	Test	0.9929	0.0048	0.0001	0.0086
E	Validation	0.9906	0.0056	0.0001	0.0108

**Table 2:** Descriptive statistics of the average COF (dimensionless) for all materials.

Material	Mean	Median	Standard deviation	Minimum	Maximum
AC	0.4603	0.4955	0.0851	0.0013	0.5587
C	0.4841	0.5151	0.1000	0.0033	0.5792
AE	0.4449	0.4725	0.0756	0.0067	0.5341
E	0.4298	0.4589	0.0970	0.0065	0.5525

## 4 Experimental Design, Materials and Methods

This section describes the experimental design and methods used to acquire the data of the wear behavior of four types of open-cell AlSi10Mg materials and open-cell AlSi10Mg-Al_2_O_3_ composites with different pore sizes under dry sliding conditions. The data include the COF as a function of time for each material, as well as the predictions of COF using the machine learning model - Extreme Gradient Boosting.

### 4.1 Testing Procedure

The testing procedure involved performing dry wear tests on four types of open-cell AlSi10Mg materials with different pore sizes and reinforcements under room temperature conditions. The materials were fabricated by using the replication method [2] and the squeeze casting [3], as described in detail in the original research article [1]. The specimens were cylindrical pins with diameter 10 mm and length 20 mm. The counterface was a hardened steel disk with hardness 62 HRC and roughness Ra 1.4 µm. Acetone was used to clean the specimens prior to and following the tests. The pin-on-disk test was conducted with four different samples, corresponding to the four types of open-cell AlSi10Mg materials. The wear experiment was repeated three times on each sample to confirm the accuracy of the results. The chemical composition of the base alloy is presented in Table 3.

**Table 3:** Chemical composition of base alloy AlSi10Mg.

Elements	Al	Si	Fe	Mn	Mg	Ti	Zn	Cu	Ni	Pb
Concentration, wt. %	rest	9.0–11.0	0.55	0.45	0.2–0.45	0.15	0.10	0.05	0.05	0.05

The choice of the parameters for designing the plan of the experiment was based on the following criteria:

• The parameters should be relevant and significant for the research objectives.
• The parameters should be available and feasible in terms of time, cost, and equipment.
• The parameters should be compatible and interactive with each other and with the materials and composites.
• The parameters should have a range and level that can cover the possible variations and scenarios of the experiment.

The chosen parameters for the experiment were:

• The type, size and content of the reinforcement: Al_2_O_3_, size of particles 300–400 µm, 5 wt.%.
• The pore size of the open-cell AlSi10Mg materials: 800–1000 µm and 1000–1200 µm.
• The linear velocity, load and sliding time of the dry wear tests: 0.5 m.s^−1^, 50 N and 420 s.
• The input data and output data for the XGBoost model: COF vs sliding time.

### 4.2 Testing Equipment

The testing equipment used for the dry wear tests was a Ducom Rotary Pin/Ball-on-Disk tribometer, model TR-20. The tribometer is equipped with a load cell that measures the friction force. The tribometer also has a data acquisition system that records the friction force and the sliding distance as a function of time. The device can perform the ASTM G99 standard test method for wear testing with the Pin-on-Disk method [4].

### 4.3 Data Acquisition

The data acquisition system of the tribometer recorded the friction force and the sliding distance as a function of time for each test. The data were stored in DWF format and can be accessed by any software for text editing or spreadsheets. The COF was determined as the ratio of the friction force to the applied load. The COF was recorded as a function of time for each specimen during the tests. The data files include three datasets for the COF as a function of time for each of the four tested materials. Four average COF as a function of time files of the three datasets of each of the four materials. One file used for the predictions of COF vs sliding time by using a XGBoost model. The data files are in DWF (raw) and XLSX (processed) format and can be opened by any text editor or spreadsheet software.

### 4.4 Coefficient of Friction Calculation

The Python script “calculate-COF-Al-Al2O3.py” performs several tasks to analyze the friction data for four different materials: AC, C, AE and E. First, it imports some libraries and modules that are needed for data analysis, plotting and file handling. Then, it reads data from several Excel files that contain friction force and time measurements for each material and assigns them to variables. Next, it calculates the COF for each dataset using a formula and a constant normal force of 50 N. After that, it computes the average coefficient of friction and time for each material by taking the mean of the three datasets. It also saves the average values as new Excel files using pandas functions. Finally, it creates two plots that show the average COF versus time for each pair of materials (AC and C, AE and E) using matplotlib functions and options. It applies a style sheet, a shadow effect, labels, legends, limits and fonts to the plots to make them more appealing and informative. It shows the plots on the screen and saves them as PNG files with high resolution.

### 4.5 Coefficient of Friction Prediction using XGBoost

The prediction code used for this study is a python script named “XGB-COF.py”. The script uses the XGBoost library to train and test a machine learning model that predicts the coefficient of friction as a function of sliding time for different materials [5,6]. The script takes as input one of the four files named “pred_COF_AC.xlsx”, “pred_COF_C.xlsx”, “pred_COF_AE.xlsx”, and “pred_COF_E.xlsx”, which contain the average coefficient of friction and sliding time data for each material. The script can only process one file at a time. The script splits the data into training and testing sets with a ratio of 80:20, and performs a 5-fold cross-validation on the training set to tune the hyperparameters of the XGBoost model. The script then evaluates the performance of the model on the testing set using four metrics: MSE, RMSE, R2, MAE. The script plots and saves the average actual vs predicted coefficient of friction as a function of time for each material in four PNG files of the four materials. The script saves the performance metrics in four TXT files, and also saves the data in four XLSX files of the actual and predicted values of the COF for two different sets (test and validation) of the four materials.

## CRediT authorship contribution statement

**Mihail Kolev:** Conceptualization, Methodology, Software, Validation, Formal analysis, Investigation, Writing – review & editing, Visualization, Supervision, Project administration, Funding acquisition. **Ludmil Drenchev:** Conceptualization, Visualization, Supervision.

## Declaration of Competing Interest

The authors declare that they have no known competing financial interests or personal relationships that could have appeared to influence the work reported in this paper.

## Data Availability

Friction coefficient data of open-cell AlSi10Mg and AlSi10Mg-Al2O3 materials with different pore sizes by pin-on-disk test and machine learning prediction (Original data) (Mendeley Data).
